# Synchronous Pleomorphic Adenoma and Warthin Tumor in the Parotid Gland: MRI Findings of Structural Compartmentalization and Quantitative T2 Signal Differences

**DOI:** 10.7759/cureus.106632

**Published:** 2026-04-08

**Authors:** Kana Suzuki, Shu Kikuta, Kensuke Yabe, Yasuyuki Nomura, Takeshi Oshima

**Affiliations:** 1 Department of Otolaryngology–Head and Neck Surgery, Nihon University School of Medicine, Tokyo, JPN

**Keywords:** mri diagnosis, parotid gland tumor, pleomorphic adenoma, synchronous tumors, t2-weighted imaging, warthin tumor

## Abstract

Synchronous tumors of different histological origins within the same parotid gland are rare and can be difficult to diagnose preoperatively, particularly when imaging findings resemble a single multilobulated mass. Pleomorphic adenoma (PA) and Warthin tumor (WT) are the two most common benign parotid tumors; however, their ipsilateral coexistence is uncommon.

We report a case of synchronous PA and WT in the superficial lobe of the left parotid gland in a 56-year-old male presenting with painless infra-auricular swelling. Magnetic resonance imaging (MRI) demonstrated a bilobed lesion. On T1-weighted imaging, the mass appeared morphologically unified. In contrast, T2-weighted imaging revealed clear structural compartmentalization between cranial and caudal components with distinct signal characteristics.

Superficial parotidectomy was performed, and histopathology demonstrated PA in the cranial component and WT in the caudal component. The combination of a sharply defined structural boundary and consistent quantitative signal differences suggested biological independence rather than intratumoral variation.

Thus, we suggest that qualitative and quantitative MRI assessment may provide useful preoperative clues for identifying histologically distinct synchronous tumors, even when the lesion appears morphologically unified.

## Introduction

Parotid gland tumors are the most common type of salivary gland tumors. Most of them are benign, with pleomorphic adenoma (PA) being the commonest, followed by Warthin tumor (WT) [1-4].

PA is characterized by epithelial and myxochondroid stromal components and often demonstrates internal architectural heterogeneity. PA typically contains a mixture of epithelial and myxochondroid stromal components, resulting in variable internal architecture and heterogeneous water content. In contrast, WT is characterized by cystic and lymphoid structures with relatively homogeneous fluid-rich components, which often produce more uniform high signal intensity on T2-weighted imaging. In contrast, WT typically occurs in older male patients, particularly smokers, and is known for its tendency toward multifocality and bilaterality [5].

Multiple tumors may arise within the parotid gland with an identical histological origin. The synchronous coexistence of tumors with different histological types within the same parotid gland is rare [6,7]. Ipsilateral synchronous PA and WT have been reported only sporadically, and preoperative diagnosis remains challenging. Distinguishing these tumor types preoperatively is clinically important because management strategies differ: PA generally requires complete surgical excision due to recurrence risk, whereas WT may be managed conservatively in selected cases.

MRI plays a central role in the preoperative evaluation of parotid tumors by enabling assessment of tumor morphology, margins, and internal signal characteristics [8,9]. However, when tumors are closely adjacent, imaging findings may mimic a multilobulated single tumor, making differentiation from intratumoral heterogeneity difficult. Thus, it remains difficult to determine whether such imaging findings represent true coexistence of independent tumors or intratumoral heterogeneity within a single lesion.

We report a rare case of synchronous PA and WT within the same parotid gland in which MRI demonstrated structural compartmentalization and distinct T2-weighted signal characteristics corresponding to two histologically independent tumor entities. In addition to conventional qualitative MRI assessment, the integration of structural compartmentalization with quantitative T2 signal analysis may provide additional diagnostic value in distinguishing synchronous tumors from intratumoral heterogeneity.

## Case presentation

A 56-year-old male presented with painless swelling in the left infra-auricular region. He had smoked 20 cigarettes per day for approximately 30 years and consumed alcohol occasionally. The swelling had been present for seven months and had gradually increased in size.

On physical examination, a well-circumscribed, elastic-hard, mobile mass measuring approximately 30 mm in diameter was palpated in the left parotid region. No facial nerve palsy or cervical lymphadenopathy was observed. The lesion was clinically interpreted as a solitary benign parotid tumor.

Fine-needle aspiration cytology of the cranial portion yielded a class III result suspicious for pleomorphic adenoma.

**Figure 1:**
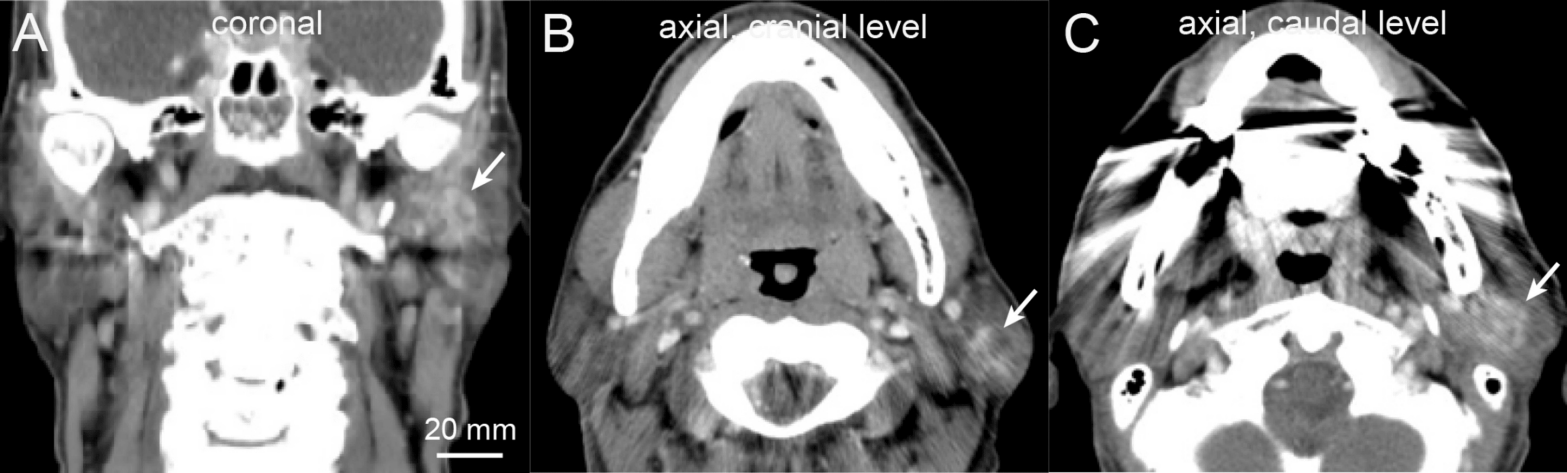
Computed tomography (CT) findings. (A) Coronal CT image. (B) Center: Axial CT image at the level of the cranial component. (C) Axial CT image at the level of the caudal component. Arrows indicate the left intraparotid tumor. The boundary between the cranial and caudal components is obscured by dental metallic artifacts.

CT demonstrated a mass within the superficial lobe of the left parotid gland; however, evaluation was limited by dental metallic artifacts (Figure 1). The boundary between the cranial and caudal components was obscured, and CT did not provide additional diagnostic information.

**Figure 2:**
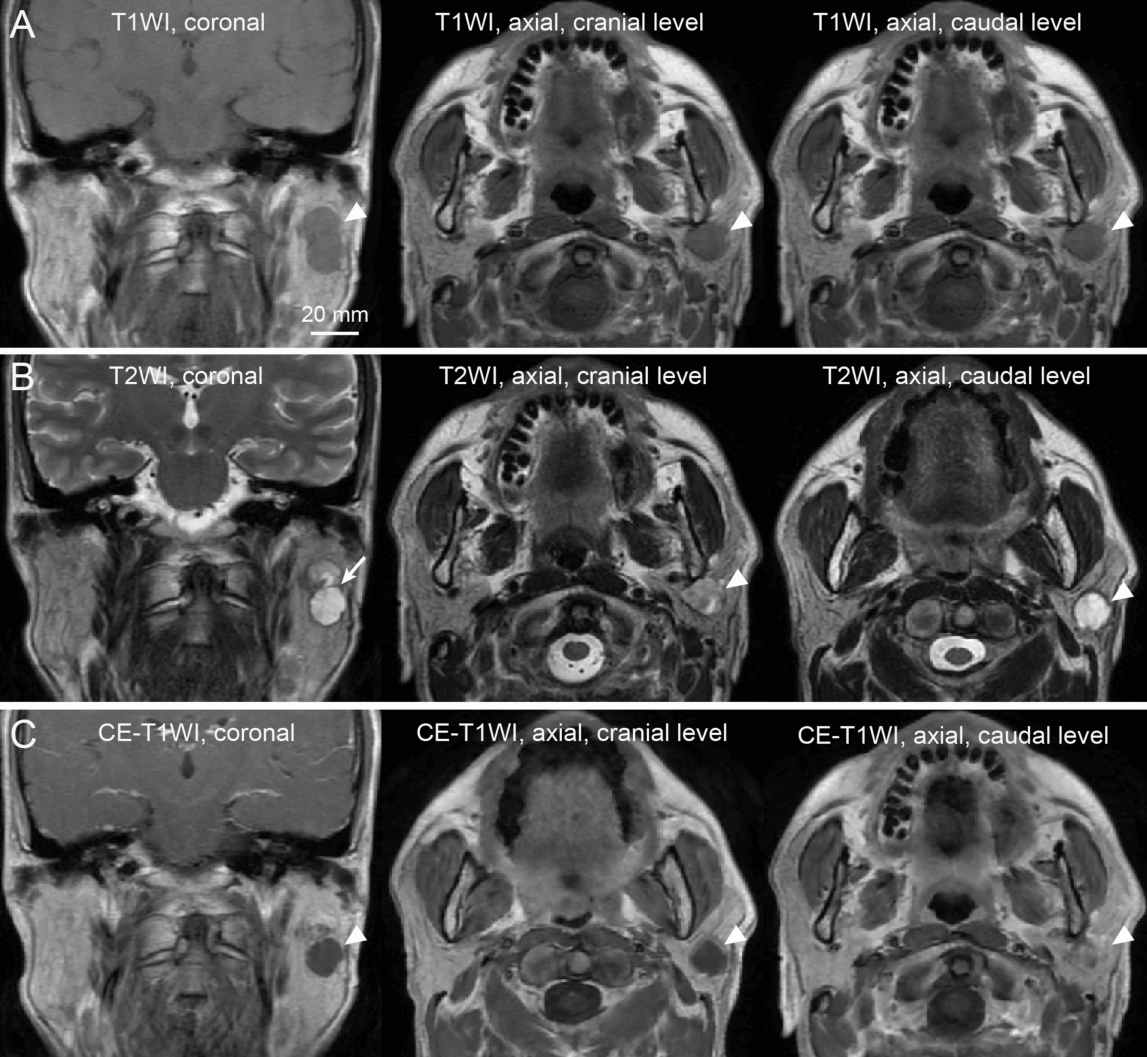
Magnetic resonance imaging (MRI) findings. (A) T1-weighted images (T1WI). Left: Coronal view. Center: Axial image at the level of the cranial component. Right: Axial image at the level of the caudal component. (B) T2-weighted images (T2WI). Left: Coronal view. Center: Axial image at the level of the cranial component. Right: Axial image at the level of the caudal component. The arrow indicates the capsule-like rim at the interface between the cranial and caudal tumor components, demonstrating structural compartmentalization. This interface represents a structural boundary suggestive of biologically distinct tumor components. (C) Contrast-enhanced T1-weighted images (CE-T1WI). Left: Coronal view. Center: Axial image at the level of the cranial component. Right: Axial image at the level of the caudal component. Arrowheads indicate the tumor in each panel.

MRI revealed a bilobed mass within the superficial lobe (Figure 2). On T1-weighted imaging, the lesion appeared morphologically as a single tumor (Figure 2A). On T2-weighted imaging, the cranial component showed heterogeneous intermediate-to-high signal intensity, whereas the caudal component exhibited homogeneous high signal intensity (Figure 2B). A clearly defined structural boundary separated the two components, and a capsule-like rim was visible at the interface, suggesting structural compartmentalization (Figure 2B). Contrast-enhanced T1-weighted imaging demonstrated minimal enhancement in both components (Figure 2C).

**Figure 3:**
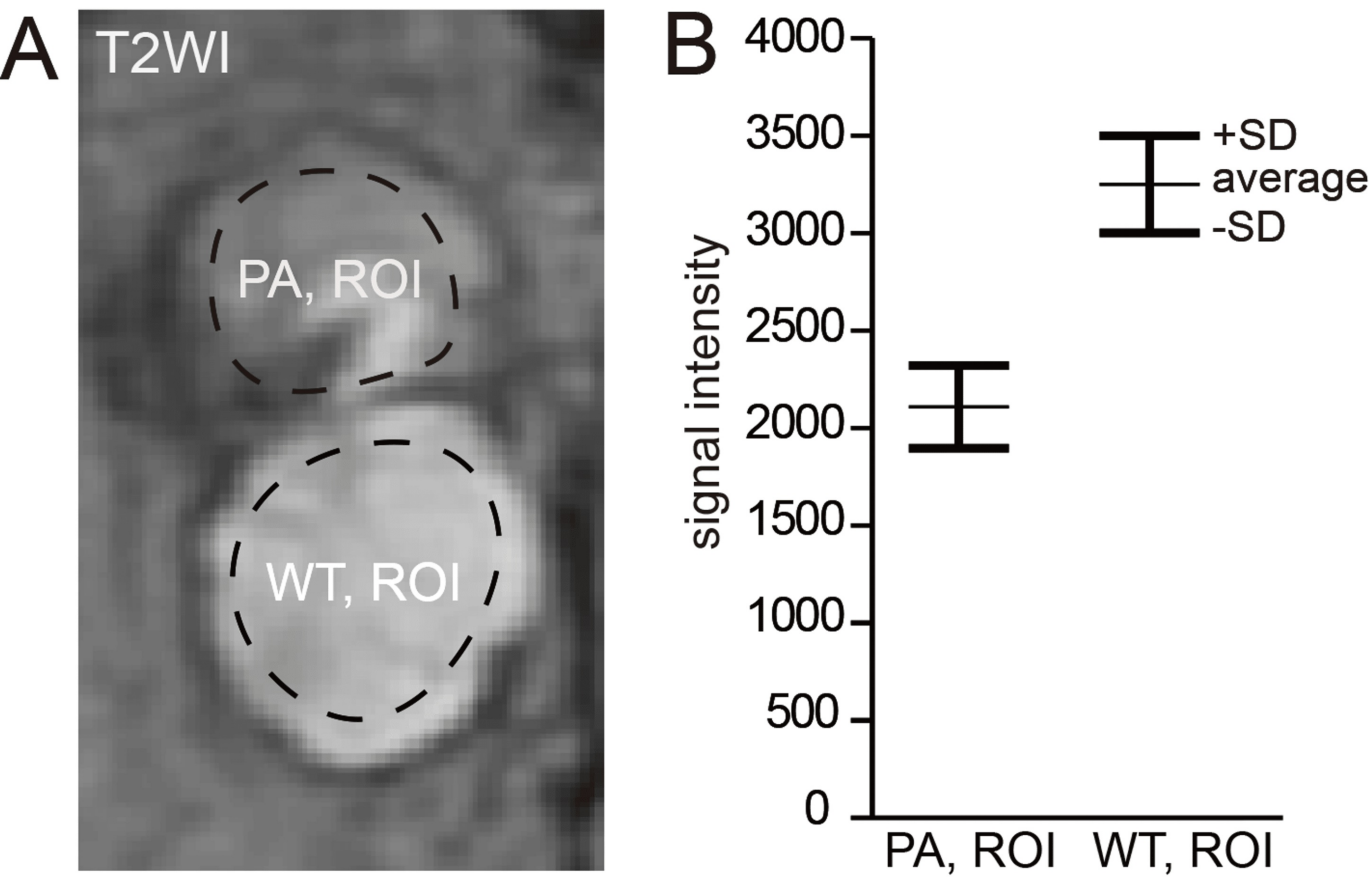
T2-weighted MRI and ROI analysis. (A) ROI placement within the solid intratumoral regions of PA and WT components. (B) Comparison of T2 signal intensities between PA-ROIs and WT-ROIs demonstrating non-overlapping mean ± SD ranges. The mean T2 signal intensity was 2115.7 ± 196.6 for PA and 3265.8 ± 261.1 for WT, indicating clearly distinct and non-overlapping distributions. The quantitative analysis supports the visual MRI impression of distinct signal characteristics between the lesions. ROI: region of interest; PA: pleomorphic adenoma; WT: Warthin tumor.

To further evaluate the observed signal disparity, region-of-interest (ROI) analysis was performed on axial T2-weighted images (Figure 3A). ROIs were manually placed within the solid intratumoral regions of PA and WT components, carefully avoiding cystic areas and minimizing partial volume effects. Measurements were obtained on the slice demonstrating the maximal cross-sectional area of each component. Signal intensity values were recorded and averaged for each lesion. The two components demonstrated distinct and non-overlapping signal intensity ranges, supporting the visual impression of consistent signal differences between the lesions (Figure 3B). Specifically, the mean T2 signal intensity of the PA component was 2115.7 ± 196.6, whereas that of the WT component was 3265.8 ± 261.1, demonstrating clearly non-overlapping signal distributions between the two lesions. These quantitative findings were intended to support the visually observed signal differences rather than to define a universal diagnostic threshold.

**Figure 4:**
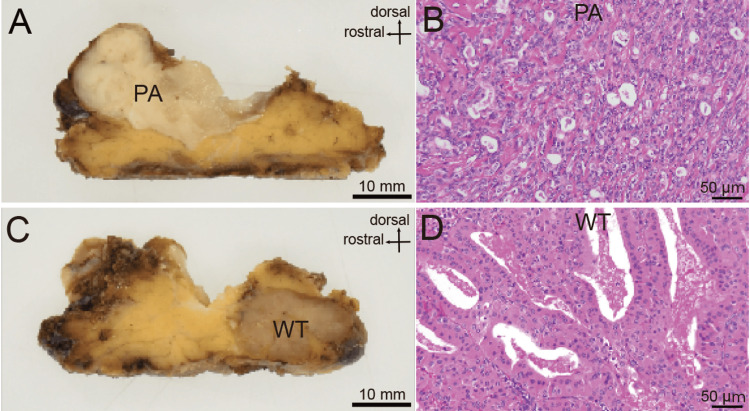
Histopathological findings. (A) Gross specimen of the cranial tumor after sectioning, showing the cut surface of the PA in the superficial lobe of the left parotid gland. (B) Histopathological image of the cranial tumor confirming pleomorphic adenoma, demonstrating epithelial and myxoid stromal components with ductal structures. (C) Gross specimen of the caudal tumor after sectioning, showing the cut surface of the WT. (D) Histopathological image of the caudal tumor confirming WT, demonstrating oncocytic epithelial layers and lymphoid stroma with follicular architecture. PA: pleomorphic adenoma; WT: Warthin tumor.

Superficial parotidectomy was performed with preservation of the facial nerve. Gross examination revealed two adjacent but clearly distinct tumor nodules within the superficial lobe (Figures 4A, 4C). Histopathological examination demonstrated pleomorphic adenoma in the cranial component (Figure 4B) and Warthin tumor in the caudal component (Figure 4D). The structural boundary identified on T2-weighted MRI corresponded to the interface between two histologically distinct tumor entities.

## Discussion

Most multilobulated or multiple parotid lesions represent multifocal tumors of identical histological origin, particularly WT, which is well recognized for its multicentric and bilateral tendencies [10,11]. In contrast, the synchronous coexistence of tumors with different histopathological origins within the same parotid gland is distinctly uncommon [6,11].

Among heterogeneous tumor combinations, PA and WT represent the most frequently described pairing [12], likely reflecting their individual prevalence rather than a specific biological association [11,13]. Their distinct microstructural compositions influence water content and stromal characteristics, thereby affecting MRI signal behavior, particularly on T2-weighted imaging, as we noticed in this case.

Preoperative recognition of synchronous PA and WT is clinically relevant. PA carries a risk of recurrence depending on capsular integrity and surgical margins [14], whereas WT is biologically indolent and may be managed conservatively in selected cases [5]. Failure to recognize heterogeneous coexistence may result in underestimation of tumor multiplicity and suboptimal surgical planning, especially if one of the tumors is malignant.

Fine-needle aspiration cytology is inherently limited in this context because sampling is localized and may not capture all tumor components. Consider taking multiple fine-needle aspirations (FNAs) from different sites if imaging shows different features, as in this case, it might help. However, it is associated with more pain and side effects for patients.

CT provides detailed anatomy of the bone but limited soft tissue anatomy, unlike MRI [8,9,15]. In this case, dental artifacts further reduced CT diagnostic value.

The key imaging feature was not merely internal heterogeneity. Heterogeneous T2-weighted signal intensity is frequently observed in PA due to stromal diversity and cystic change, and does not in itself indicate multiple tumor types. What distinguished this lesion was the coexistence of two findings: a sharply demarcated structural boundary dividing the mass into compartmentalized units and consistently distinct quantitative T2-weighted signal characteristics between these units. MRI offers a wide variety of imaging techniques beyond conventional T1- and T2-weighted imaging. While T2-weighted imaging (with quantitative ROI analysis) was particularly useful in this case for demonstrating structural compartmentalization and tissue-specific signal differences, other sequences could provide complementary preoperative insights into synchronous parotid tumors. In the present case, the mean T2 signal intensity values of the two lesions were clearly separated, with no overlap between their mean ± SD ranges (PA: 2115.7 ± 196.6; WT: 3265.8 ± 261.1), further supporting the interpretation of two biologically distinct tumor entities rather than intratumoral heterogeneity. From a practical perspective, clinicians may suspect synchronous tumors when the following features are present: (1) a sharply defined structural boundary between adjacent components, (2) consistent internal signal differences, and (3) non-overlapping quantitative T2 signal intensity profiles. Although not definitive individually, their combination may provide a useful clinical clue.

Such structural discontinuity implies biological independence rather than intratumoral variation. While PA may demonstrate internal variability, it generally preserves architectural continuity. In contrast, a well-defined interface separating regions with consistently different signal profiles is more compatible with two independent neoplastic processes. Although internal heterogeneity or capsule-like features may individually occur in benign tumors, their combined presence may provide an imaging clue to histologically distinct synchronous tumor components. In recent years, there have been reports of machine learning methods that distinguish between different tissue types based on image features in addition to intensity; therefore, it might be worthwhile to include a brief discussion of this.

This report has limitations. It describes a single case, and the findings may not be generalizable. Quantitative assessment was exploratory and performed within routine clinical imaging rather than a standardized research protocol. In addition, because ROI measurements were performed manually in a single case, reproducibility and interobserver variability could not be assessed. Nevertheless, combined structural and quantitative MRI evaluation may assist in distinguishing true tumor coexistence from conventional intratumoral heterogeneity.

## Conclusions

Synchronous PA and WT within the same parotid gland are rare and are challenging to diagnose. Integrated qualitative and quantitative MRI evaluation has a role in identifying synchronous tumors. Followed by surgical excision for definitive diagnosis. In this case, both structural compartmentalization and distinct quantitative T2-weighted signal confirmed the presence of different synchronous tumors within the same gland. These findings should be interpreted cautiously, given the single-case nature of this report, and further validation in larger cohorts is warranted.
